# A Confirmatory Factor Analysis of Facets of Psychological Flexibility in a Sample of People Seeking Treatment for Chronic Pain

**DOI:** 10.1007/s12160-015-9752-x

**Published:** 2015-11-25

**Authors:** Whitney Scott, Lance M. McCracken, Sam Norton

**Affiliations:** Health Psychology Section, Institute of Psychiatry, Psychology, and Neuroscience, King’s College London, Guy’s Campus, London, SE1 9RT UK; INPUT Pain Unit, Guy’s and St. Thomas’ NHS Foundation Trust, London, UK

**Keywords:** Chronic pain, Psychological flexibility, Confirmatory factor analysis

## Abstract

**Background:**

Evidence supports the validity of individual components of the psychological flexibility model in the context of chronic pain. However, there is a need to test the inter-relationships amongst measures of individual components of psychological flexibility in a more integrative manner. In particular, research is needed to examine whether a model with discrete facets as proposed is indeed reflected in data from currently used assessment measures in people with chronic pain.

**Purpose:**

This cross-sectional study investigated the underlying structure of measures of processes of psychological flexibility amongst individuals with chronic pain and the associations between this measurement model and patient functioning.

**Methods:**

Five-hundred and seventy-three adults with chronic pain completed measures of pain, physical and social functioning, mental health, depression and processes of psychological flexibility, including acceptance, cognitive defusion, decentering and committed action. Confirmatory factor analyses tested lower-order, higher-order and bifactor models to examine the structure of psychological flexibility process measures.

**Results:**

A single general factor reflecting openness explained variability in items across all of the psychological flexibility process measures. In addition to this general factor, distinct decentering and committed action group factors emerged in the data. As expected, the general factor was strongly correlated with measures of social functioning, mental health and depression.

**Conclusions:**

Future research is needed to determine the most useful means by which the presence of the general factor can be reflected in the measurement and theory of psychological flexibility.

## Introduction

The importance of psychological factors in the development and maintenance of chronic pain and associated disability is now virtually established. For the past 30 years, cognitive-behavioural models of pain and disability have been largely successful in guiding psychological research into chronic pain and contributing to this achievement [1, 2]. These models have also proven useful in the development of psychologically based treatment approaches, primarily cognitive behavioural therapy, for which there is now good evidence in patients with chronic pain [3].

More recently, there is growing interest in Acceptance and Commitment Therapy (ACT) and its underlying theoretical model as a means to promote a next generation of treatment developments in chronic pain [4, 5]. Briefly, ACT for chronic pain aims to help individuals disengage from unsuccessful efforts to control or avoid pain and instead engage in efforts to reach positive goals and follow personal values. The somewhat counterintuitive aim in ACT is for this latter engagement to occur in the presence of potentially interfering psychological experiences, such as thoughts and feelings, yet without these experiences functioning as barriers to this engagement [5, 6]. Accumulating evidence, including more than 10 published, randomised controlled trials and numerous uncontrolled trials, supports the efficacy and effectiveness of ACT for individuals with chronic pain [7, 8].

ACT is theoretically rooted in the psychological flexibility model [4, 9, 10]. Psychological flexibility is the capacity to persist with and change behaviour in a manner that incorporates conscious and open contact with thoughts and feelings and that is consistent with one’s values and goals [4]. Psychological flexibility is suggested to comprise the following six related processes: (1) *acceptance*, a willingness to experience difficult emotions; (2) *cognitive defusion*, a loosening of the dominance of thoughts over experience and actions; (3) *flexible present-focussed awareness*, purposeful, non-judgmental attention to present experiences; (4) *self-as-context*, a perspective in which there is a distinction between the person having an experience and the experiences themselves; (5) *values-based action*, behaving in ways consistent with one’s chosen values; and (6) *committed action*, flexible persistence in values-based and goal-directed behaviour. These processes have recently been conceptualised more succinctly as the capacity for behaviour that is ‘open, aware, and engaged’, reflecting acceptance and defusion, present-focussed awareness and self-as-context, and values-based and committed action, respectively [11].

Growing evidence supports the validity and predictive utility of components of psychological flexibility in chronic pain [12]. Research to date has predominantly focussed on acceptance, values-based action and present-focussed awareness, with findings linking these processes to improved physical, social and emotional functioning [13–17]. More recently, measures of cognitive defusion and committed action have been validated in the context of chronic pain [18–20]. A validated, comprehensive measure of self-as-context is not yet available in the literature. However, whilst not originally developed within the psychological flexibility framework, a measure of decentering, which includes content tapping cognitive defusion, awareness and self-as-context, has recently been validated for use amongst patients with chronic pain [21]. Studies employing these currently available measures have shown that the processes they reflect are associated with measures of daily functioning, particularly emotional and social functioning, in people with chronic pain [18–21].

In light of accumulating support for the validity of measures assessing individual components of the psychological flexibility model, there is a need to test the inter-relationships amongst measures of component processes in a more integrative manner, as a means to potentially simplify or improve measurement methods. To this end, Vowles and colleagues recently undertook the first comprehensive examination of the factor structure of measures of psychological flexibility processes and the association between this measurement model and patient functioning in chronic pain [22]. Exploratory factor analyses were conducted on pre-treatment assessment data from 274 patients with chronic pain who completed self-report measures of a number of facets related to psychological flexibility. These analyses resulted in a three-factor model, which the authors labelled as ‘acceptance/defusion’, ‘values/committed action’, and ‘moment-to-moment awareness/self-as-context’ based on the pattern of item loadings. Although a three-factor structure emerged from the data, these factor labels clearly reflect the original conceptualisation of psychological flexibility in terms of six inter-related processes as outlined above. Each of these factors were moderately to strongly inter-correlated and significantly correlated with pain intensity, daily functioning and distress in a structural equation model [22].

The findings by Vowles and colleagues are an important first step towards understanding the structure of related measures assessing individual processes of psychological flexibility in chronic pain. However, replication is required and several questions remain. For instance, although the authors labelled one of their factors as values/committed action, none of their measures directly assessed committed action. Therefore, models explicitly incorporating this aspect of psychological flexibility are needed.

Additionally, research has not tested whether a general factor explains variability in items across measures of these processes using an appropriate approach, such as bifactor modelling, which can simultaneously evaluate the unidimensionality and multidimensionality of a group of items [23]. This is despite strong indications for the presence of a general factor across measures of psychological flexibility processes. For instance, the study by Vowles et al. reported eigenvalues indicating that 46 % of the total item variance was explained by the first unrotated factor, excluded two measures of psychological flexibility due to cross loadings and identified moderate to strong inter-correlations amongst the three factors reflecting the processes [22]. These observations point towards a potentially hierarchical latent structure and the potential presence of a general factor [24, 25].

Extending the analysis of Vowles and colleagues to more robustly consider the presence of a general factor across measures of psychological flexibility will provide greater understanding of the structure of these measures. In turn, this understanding may lead to improved psychometric assessment of this construct and, as data accumulate, theoretical refinements. For example, the presence of a general factor across existing measures might suggest that refinements to these measures are needed to better reflect the distinct processes, as currently conceptualised in the model. Alternately, if accumulating data indicate that a single factor clearly underlies each process of psychological flexibility, revisions to the current multipart structure of the psychological flexibility model might be warranted.

The purpose of this study was to investigate the structure of measures assessing processes of psychological flexibility. Individuals with pain attending an interdisciplinary ACT-based treatment programme completed measures of pain, physical and social functioning, mental health, depression and psychological flexibility processes as part of their standard pre-treatment assessment. In particular, measures of acceptance, cognitive defusion, decentering and committed action were included in the assessment battery as they reflect the ‘open, aware and engaged’ components of psychological flexibility and emerging data support the validity of these measures in chronic pain, as discussed. Confirmatory factor analyses tested several models to examine the potentially hierarchical latent structure of process measures associated with psychological flexibility. Correlations were computed to examine the associations between the bifactor confirmatory factor analysis model and patient-reported outcomes. It was predicted that analyses would demonstrate interpretable subcomponents and a unitary general factor from the measures examined and that these would show significant associations with measures of key aspects of daily functioning.

## Methods

### Participants

Participants were consecutive referrals to an adult, 4-week, residential, interdisciplinary pain management programme at a comprehensive pain treatment centre in central London, UK, who began treatment between January 2012 and April 2014. Participants were selected for the treatment if they had pain of greater than 3 months duration that was associated with significant levels of distress and disability, and they were deemed likely to benefit from the programme based on assessment by a specialist physiotherapist and psychologist.

This was a cross-sectional, observational study that utilised data collected from a standardised battery of clinical assessment measures (described below). Clinical assessment procedures were updated after April 2014 and, as such, this determined the end point for the present study. Thus, the sample size was not pre-determined; all participants during the time period described were included in the present sample. Five-hundred and ninety patients initially began treatment. Of these, 15 did not consent to have their data used for research purposes. Two had missing data on all of the measures related to psychological flexibility and were excluded from the analyses. Thus, all analyses were run on the sample of 573 (380 women and 193 men). The most common pain sites reported were pain in the lower back (42.7 % of patients) and generalised pain (20.8 %). The mean age of participants was 46.73 years (SD = 11.29 years) and a mean pain duration of 152.86 months (SD = 130.34 months). Further background details are included in Table 1.

**Table 1:** Patient demographics

	Mean (SD) or *n* (%)
Age at assessment	46.73 (11.29)
Pain duration (in months)	152.86 (130.34)
Years of education	13.14 (4.07)
Primary pain site	
Head, face or mouth	15 (2.6 %)
Neck region	42 (7.3 %)
Upper shoulder/limbs	42 (7.3 %)
Chest region	7 (1.2 %)
Abdominal region	12 (2.1 %)
Lower back/spine	244 (42.7 %)
Lower limbs	75 (13.1 %)
Pelvic region	7 (1.2 %)
Anal/genital	9 (1.6 %)
Generalised	119 (20.8 %)
Missing	1 (0.2 %)
Sex	
Male	193 (33.7 %)
Female	380 (66.3 %)
Ethnic group	
White	408 (71.2 %)
Black	94 (16.4 %)
Asian	42 (7.3 %)
Mixed	24 (4.2 %)
Other/missing	5 (0.9 %)
Work status	
Employed	156 (27.2 %)
Unemployed due to pain	303 (52.9 %)
Unemployed for other reason	16 (2.8 %)
Other/missing (retired, student, etc.)	98 (17.1 %)

### Procedure

On the first day of the treatment course patients were asked to complete standard baseline assessment material. This included self-report measures of pain intensity, physical and social functioning, mental health, depression and measures assessing processes of psychological flexibility. To limit potential biases associated with poor validity and reliability, all self-report measures used for this study were previously validated and shown to have strong psychometric properties. Patients also provided background information, including their sex, age, ethnicity, pain location and duration, living situation, years of education and work status. All participants provided written informed consent to have their data used for the purpose of research. The research database and study were granted local ethics and National Health Service Research and Development approvals prior to commencing data collection. The data used here included data from the pre-treatment baseline assessment only.

### Measures

#### Pain Intensity

Participants rated their average pain in the past week on a standard scale from 0 (no pain) to 10 (extremely intense pain).

#### Physical and Social Functioning

The Short-Form Health Survey (SF-36) [26] is a standardised measure of health status consisting of 36 items. The SF-36 yields eight subscale scores assessing various domains of life functioning. The physical and social functioning and mental health subscales were used for the purpose of the present study. Higher scores indicate better functioning in these domains. The SF-36 has been validated and is widely used as a measure of health status and function amongst patients with chronic pain [27].

#### Depression

The depression module of the Patient Health Questionnaire PHQ-9 [28] was used to measure the severity of patients’ symptoms of depression based on standard DSM-IV diagnostic criteria. On this measure, patients report on the frequency with which they experience nine different symptoms from 0 (not at all) to 3 (nearly every day). One additional item assesses the impact of these symptoms on work, home and social activities. The total score of the first nine items reflects the severity of depression, with higher scores indicating greater severity. The PHQ-9 has been well validated amongst patients with chronic health conditions [28].

#### Acceptance

Acceptance can be defined as the opposite of experiential avoidance, or an unwillingness to experience unwanted feelings and emotions, particularly when this pattern of behaviour is inconsistent with one’s goals and values [10]. The seven-item Acceptance and Action Questionnaire (AAQ) was used as a general measure of acceptance for this study [29]. Each item is rated on a seven-point scale from 1 (never true) to 7 (always true). All items on the AAQ are keyed in the negative direction and thus have to be reversed before producing the total score. Once reverse scored, higher scores reflect greater acceptance. Examples include, ‘*I’m afraid of my feelings*’ and ‘*Worries get in the way of my success*’. Analyses of the AAQ provide good support for its internal consistency, temporal stability and construct validity [29]. The reliability and validity of the AAQ for use in patients with chronic pain have also previously been reported [30]. In the present sample, total scores on the AAQ ranged from 7 to 49. Cronbach’s alpha in this sample was 0.92, indicating excellent internal consistency.

#### Cognitive Defusion

Cognitive defusion is the process of experiencing a distinction between thoughts and the situations, events or people to which they refer. It also describes a loosening of the dominance of thoughts over experience and actions [10]. The seven-item version of the Cognitive Fusion Questionnaire (CFQ) was used to measure cognitive defusion [31]. On this measure, participants are asked to rate items on a seven-point scale with the end points 1 (never true) and 7 (always true). All items of the CFQ are keyed in the direction of cognitive fusion. Therefore, for the purpose of this study, items were reverse scored so that higher scores reflect greater defusion. Examples include, ‘*My thoughts cause me distress or emotional pain*’ and ‘*I get so caught up in my thoughts that I am unable to do the things that I most want to do*’. Recent findings support the reliability and validity of the CFQ for use amongst individuals with chronic pain [18]. Total scores on the CFQ in the current sample ranged from 7 to 49. Cronbach’s alpha in this sample was 0.93, indicating excellent internal consistency.

#### Decentering

Decentering is defined as the ability to observe one’s thoughts and feelings as temporary objective events in the mind, rather than as ‘true’ reflections of the self or one’s circumstances [32]. The 12-item decentering subscale of the Experiences Questionnaire was used to assess decentering [21, 33]. This measure asks individuals to rate each item on a five-point scale ranging from 1 to 5, with the following anchors: never, rarely, sometimes, often or all the time. Higher scores on this measure indicate greater decentering. Item examples include, ‘*I can separate myself from my thoughts and feelings*’ and ‘*I can actually see that I am not my thoughts*’. Data support the internal reliability of the decentering subscale amongst individuals with chronic pain and suggest that decentering uniquely contributes to outcomes such as mental health and social functioning [21]. In the present sample, total scores on the decentering subscale ranged from 12 to 59. Cronbach’s alpha in this sample was 0.85, indicating good internal consistency.

#### Committed Action

Committed action can be defined as flexible persistence in goal-directed behaviour [10]. Committed action was assessed with the shortened eight-item version of the Committed Action Questionnaire (CAQ-8) [20]. Respondents are asked to rate the extent to which each of the items applies to them on a seven-point scale ranging from 0 (never true) to 6 (always true). The item pool includes four positively phrased items, including, ‘*I can remain committed to my goals even when there are times that I fail to reach them*’ and four negatively phrased items, ‘*I find it difficult to carry on with an activity unless I experience that it is successful*’. Previous findings suggest that these positive and negative items load onto two separate factors, thus supporting the use of two subscales [20]. The positive and negative subscales are presumed to not just reflect an item keying issue. Instead, these subscales together provide a more complete understanding of behaviours that individuals both are and are not engaging in, flexibly pursuing goals even when difficulties arise *and* rigidly pursuing goals or failing to pursue goals in the face of challenges (these items negatively keyed). For the purpose of the present study, these two subscales were retained. To maintain consistency with the scoring of measures in the direction of psychological flexibility, items from the negatively keyed subscale were reverse scored so that higher scores reflect greater committed action. Data from patients with chronic pain support the reliability, validity and multidimensionality of the CAQ-8 [20]. For both CAQ-8 subscales, the total scores ranged from 0 to 24 in the current sample. Cronbach’s alpha were 0.86 and 0.77 in this sample for the first and second subscales, respectively, indicating good internal consistency.

### Data Analysis

**Fig. 1:**
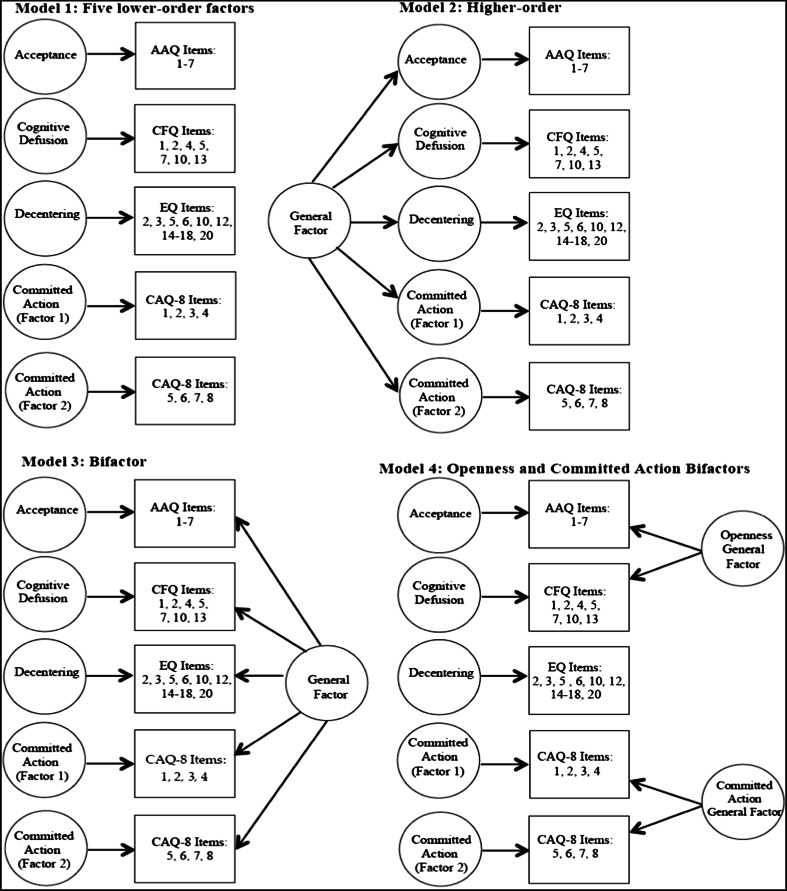
Schematic of confirmatory factor analysis models tested. *Note AAQ* Acceptance and Action Questionnaire, *CFQ* Cognitive Fusion Questionnaire, *EQ* Experience Questionnaire and *CAQ* Committed Action Questionnaire. AAQ, CFQ, and CAQ-factor 2 items were reverse scored. Item numbers reported for the CFQ and EQ are in relation to their respective full-length measures

Means and standard deviations were computed for psychological flexibility process measures and patient-reported outcomes. Confirmatory factor analysis of the psychological flexibility process measures was conducted using MPlus 7.11 [34]. Preliminary analyses confirmed adequate fit of the expected latent structures of the five scales included in the hierarchical confirmatory factor analysis models (results not shown). A number of lower-order, higher-order and bifactor models were estimated using the robust weighted least squares method. These models are summarised schematically in Fig. 1. Briefly, the higher-order approach identifies the variance of each lower-order latent factor accounted for by a general factor. In contrast, bifactor modelling parses the variance for each item into components explained by the general factor and group factors relating to common item response variance not explained by the general factor. Item saturation describes when the majority of the common variance of a set of questionnaire items is accounted for by a general factor. In other words, item saturation occurs when a set of items all appear to measure the same construct.

First, a lower-order five-factor model was tested, in which all items coming from the same questionnaire loaded onto the same factor (model 1). For this model, items from the Acceptance and Action Questionnaire, Cognitive Fusion Questionnaire, and the decentering subscale of the Experiences Questionnaire were loaded onto three separate factors, reflecting acceptance, cognitive defusion and decentering, respectively. Items from the Committed Action Questionnaire were loaded onto two factors, reflecting the multidimensionality of this measure suggested in a previous validation study [20]. Next, a higher-order model was tested to examine the extent to which a higher-order factor accounts for the associations between the lower-order factors of acceptance, cognitive defusion, decentering and the two committed action factors (model 2). A bifactor model was also tested to examine whether the variance in all of the items across the questionnaires could be explained by a single underlying factor (model 3). Lastly, we tested a model with two bifactor measurement models, an ‘openness’ general factor for acceptance and defusion items, a ‘committed action’ general factor for all of the committed action items and a lower-order decentering factor (model 4); this final model closely relates to the three-factor structure examined by Vowles and colleagues [22] and the re-conceptualisation of psychological flexibility in terms of processes of open, aware and engaged [11]. The specific set of measures used in our study tapped into aspects of these ‘aware’ and ‘engaged’ components but are not comprehensive measures of these. Therefore, it was decided to label these factors according to the specific measurement tool used to more accurately reflect the item content.

For each model, the chi-squared statistic was computed as one conventional measure of model fit. However, given the sensitivity of the chi-squared statistic to large sample sizes, several additional fit indices were evaluated to determine the adequacy of the models tested. Assessment of goodness of fit of these models was based on the following standard structural equation modelling cutoff criteria: root mean square error of approximation (RMSEA) ≤ 0.08, comparative fit index (CFI) ≥ 0.95 and Tucker-Lewis index (TLI) ≥ 0.95 [35]. The most appropriate model was selected on the basis of these fit statistics, inspection of the overall pattern of factor loadings and correlations between factors within the models. Correlations were then computed to examine the associations between the factors within this model and patient-reported outcomes.

## Results

Means and standard deviations for all study variables are presented in Table 2. Mean scores on the measures of pain intensity, depression and daily functioning are comparable (i.e. within one standard deviation) to those reported in previous studies of patients with chronic pain [36].

**Table 2:** Means and standard deviations of the total scores for the study variables

Variable	Mean (SD)
Acceptance	24.33 (11. 01)
Cognitive defusion	26.18 (11.15)
Decentering	36.70 (8.12)
Committed action subscale 1	14.51 (5.26)
Committed action subscale 2	12.56 (5.32)
Depression	16.86 (6.19)
Physical functioning	22.73 (18.18)
Social functioning	33.33 (22.98)
Mental health	42.75 (23.14)
Pain intensity	7.67 (1.63)

### Confirmatory Factor Analysis

Confirmatory factor analysis was applied to all 34 items from the four psychological flexibility process questionnaires (summarised in Table 3). Several competing models were considered in these analyses. Model 1, including five lower-order factors, showed acceptable fit (chi-square (517) = 1829.92, *p* < 0.001; RMSEA = 0.07; CFI = 0.96; TLI = 0.95). The correlations between the five factors ranged from *r* = 0.42 (decentering with committed action of factor 2) to 0.90 (acceptance with cognitive defusion), all *p*-values < 0.001. The high magnitude of the inter-correlations amongst these factors potentially suggests the presence of a general factor, indicating that further examination of hierarchical factor models was warranted.

**Table 3:** Summary of confirmatory factor analysis results

Model	Number of free parameters	*χ* ^2^	*df*	RMSEA	90 % CI	CFI	TLI
1. Five factor	224	1829.92*	517	0.07	0.06–0.07	0.96	0.95
2. Higher-order factor	219	2326.03*	522	0.08	0.07–0.08	0.94	0.94
3. Bifactor (all items)	248	2075.68*	493	0.08	0.07–0.08	0.95	0.94
4. Openness bifactor, committed action bifactor and decentering factor	239	2022.31*	502	0.07	0.07–0.08	0.95	0.94

*RMSEA* root mean square error of approximation, *CI* confidence interval, *CFI* comparative fit index, *TLI* Tucker-Lewis index

*p < 0.001

In model 2, a higher-order factor model was tested. The higher-order factor model showed similar although slightly poorer fit, as compared to the lower-order model (chi-square (522) = 2326.03, *p* < 0.001; RMSEA = 0.08; CFI = 0.94; TLI = 0.94). In this analysis, each lower-order factor loaded significantly (all *p* values < 0.001) onto the higher-order factor: acceptance (β = 0.92), cognitive defusion (β = 0.95), decentering (β = 0.58), committed action factor 1 (β = 0.56) and committed action factor 2 (β = 0.80). The higher-order factor was mainly driven by acceptance and cognitive defusion and explained more than 80 % of the variance in each of these two factors.

A bifactor model (model 3) was tested to examine whether variance across all items was accounted for by a general underlying factor. This model showed comparable fit to the higher-order model (chi-square (493) = 2075.68, *p* < 0.001; RMSEA = 0.08; CFI = 0.95; TLI = 0.94). Examination of the factor loadings of individual items onto the general factor indicated that acceptance and cognitive defusion items had the strongest loadings (range β = 0.62 to 0.89), followed by committed action items (range β = 0.42 to 0.75) and decentering items (0.09 to 0.51) (Table 4). The general factor explained 62.0 % of the common variance between items and strongly influenced the scores on the individual scales (omegahierarchical = 0.82). After parsing the variance explained by the general factor, the acceptance and cognitive defusion factors explained just 4.8 and 3.3 % of the remaining common item variance. Further supporting general factor saturation, the loadings of acceptance and defusion items onto their respective group factors were generally low (range β = 0.04–0.50) and weaker than the loadings of these items onto the general factor. Decentering items were the least saturated by the general factor. After removing the variance accounted for by the general factor, the decentering group factor explained 16.3 % of the common item variance. Overall, decentering items loaded more strongly onto the decentering group factor (range β = 0.41–0.61) than onto the general factor. After parsing the variance explained by the general factor, the first and second committed action factors explained 9.4 and 4.1 % of common item variance, respectively. Committed action items had comparable loadings (all >0.35) on both the general and the first and second committed action group factors.

**Table 4:** Standardised factor loadings for general factor model (model 3)

Item	General factor	Acceptance	Defusion	Decentering	Committed action, positive	Committed action, negative
AAQ1	0.62**	0.48**				
AAQ2	0.76**	0.38**				
AAQ3	0.82**	0.33**				
AAQ4	0.68**	0.50**				
AAQ5	0.83**	0.25**				
AAQ6	0.69**	0.27**				
AAQ7	0.83**	0.19**				
CFQ1	0.88**		0.06			
CFQ2	0.89**		0.04			
CFQ4	0.72**		0.42**			
CFQ5	0.84**		0.33**			
CFQ7	0.70**		0.37**			
CFQ10	0.80**		0.40**			
CFQ13	0.73**		0.20**			
EQ2	0.09*			0.50**		
EQ3	0.42**			0.46**		
EQ5	0.35**			0.56**		
EQ6	0.34**			0.47**		
EQ10	0.49**			0.47**		
EQ12	0.25**			0.41**		
EQ14	0.51**			0.51**		
EQ15	0.42**			0.48**		
EQ16	0.23**			0.51**		
EQ17	0.29**			0.54**		
EQ18	0.12*			0.61**		
EQ20	0.31**			0.53**		
CAQ1	0.44**				0.68**	
CAQ2	0.43**				0.66**	
CAQ3	0.47**				0.71**	
CAQ4	0.47**				0.60**	
CAQ5	0.45**					0.37**
CAQ6	0.59**					0.49**
CAQ7	0.75**					0.44**
CAQ8	0.42**					0.45**

AAQ, CFQ and CAQ-factor 2 items were reverse scored prior to analysis. Item numbers reported for the CFQ and EQ are in relation to their respective full-length measures

*AAQ* Acceptance and Action Questionnaire, *CFQ* Cognitive Fusion Questionnaire, *EQ* Experience Questionnaire, *CAQ* Committed Action Questionnaire

*p < 0.05; **p < 0.001

Model 4 tested a bifactor model with a general openness factor, a general committed action factor and a lower-order decentering factor, similar to the three-factor structure identified by Vowles et al. [22]. This model showed comparable fit to the bifactor model which included a single general factor (chi-square (502) = 2022.31, *p* < 0.001; RMSEA = 0.07; CFI = 0.95; TLI = 0.94). All of the acceptance and defusion items loaded strongly onto the openness bifactor (range β = 0.62 to 0.90). All of the committed action items loaded strongly onto the committed action bifactor (range β = 0.45 to 0.82). Decentering items all loaded strongly onto the lower-order decentering factor (range β = 0.34 to 0.79). The general openness factor explained 86.1 % of the common item variance of the acceptance and cognitive defusion items. In contrast, controlling for the openness factor, the separate acceptance and cognitive defusion group factors explained only 4.6 and 2.7 % of the remaining item variance, respectively. The general committed action factor explained 52.5 % of the common item variance of the committed action items. Controlling for the general committed action factor, the first and second committed action group factors explained 8.6 and 3.2 % of the remaining item variance, respectively. However, the correlation between the two general factors in this model was high (*r* = 0.87), suggesting that the general committed action and openness factors are not independent. The high correlation between these general factors and the comparable fit of the more parsimonious model 3 suggest that a model with a single general factor provides the most appropriate empirical representation of the relations between individual item responses to the four questionnaires considered.

### Correlations Between Factors Within the General Factor Model and Patient-Reported Outcomes

Table 5 reports the results of correlation analyses examining the associations between factors within the general factor model (model 3) and patient-reported outcomes. As can be seen, the general factor was significantly positively correlated with social functioning and mental health and negatively correlated with depression and pain intensity. In general, the correlations between the general factor and patient-reported outcomes were moderate to large in magnitude. In comparison, once the variance explained by the general factor was removed, the correlations between the group factors and patient outcomes were generally non-existent or small. Because of the level of saturation in item response by the general factor, the correlations between the group factors and patients outcomes are generally not interpretable.

**Table 5:** Correlations between the general and group factors (model 3) with patient-reported outcomes

	General factor	Acceptance	Cognitive defusion	Decentering	Committed action factor 1	Committed action factor 2
Social function	0.43**	0.08	−0.04	0.13**	0.03	0.02
Physical function	0.07	0.14*	0.18**	−0.05	−0.01	0.05
Mental health	0.71**	−0.08*	−0.15**	0.15**	0.02	0.13**
Depression	−0.66**	−0.06	−0.01	−0.17**	−0.01	0.00
Average pain	−0.21**	−0.13*	0.13*	0.08	0.14*	0.06

Group factor correlations reflect associations between group factors and patient outcomes after removing the variance accounted for by the general factor

*p < 0.05; **p ≤ 0.001

For comparison, Table 6 displays the correlations between the five lower-order factors and patient-reported outcomes from model 1, where the variance explained by the general factor is not removed. The magnitude of correlations between the lower-order acceptance and defusion factors and patient-reported outcomes (model 1) is nearly identical to the magnitude of those observed between the general factor and patient outcomes (model 3). This pattern of correlations across the two models further demonstrates saturation of the acceptance and cognitive defusion items by the general factor. The correlations between the lower-order decentering factor, committed action factors 1 and 2 and patient outcomes (model 1) were also of a similar albeit slightly weaker magnitude, to the correlations between the general factor and patient outcomes (model 3). The slight discrepancy in these correlations reflects the relatively weaker loading of decentering and committed action items onto the general factor and the simultaneous loading of these items onto their respective group factors in the bifactor model.

**Table 6:** Correlations amongst lower-order factors (model 1) and patient-reported outcomes

	Acceptance	Cognitive defusion	Decentering	Committed action factor 1	Committed action factor 2
Social function	0.43**	0.41**	0.36**	0.27**	0.36**
Physical function	0.12*	0.02	0.00	0.03	0.09
Mental health	0.60**	0.61**	0.54**	0.42**	0.50**
Depression symptoms	−0.63**	−0.62**	−0.53**	−0.38**	−0.52**
Average pain	−0.23**	−0.16**	−0.06	0.00	−0.14*

*p < 0.05; **p ≤ 0.001

## Discussion

The purpose of this study was to investigate the structure of measures assessing processes of psychological flexibility in individuals with chronic pain attending a treatment programme based on Acceptance and Commitment Therapy. To this end, four competing factor models were tested using confirmatory factor analysis of pre-treatment assessment data on measures of acceptance, cognitive defusion, decentering and committed action. The results indicated that model fit was adequate and comparable for all models tested. Despite similar fit across models, the moderate to strong correlations amongst the lower-order factors and between the openness and committed action general factors suggested that the questionnaire items are saturated by the presence of a single general factor. The comparable magnitude of the correlations between lower-order factors and the general factor with patient outcomes provides additional evidence of item saturation by the general factor.

The pattern of factor loadings in the bifactor model indicated that the general factor was dominated by acceptance and defusion items. Decentering and committed action items were also reflected in the general factor, albeit to a lesser degree. Although not explicitly measures of acceptance or cognitive defusion, the decentering and committed action subscales do contain content related to individuals’ willingness to experience difficult thoughts and feelings. Thus, the general factor appears to reflect the process of openness across the measures used here. Committed action and decentering items also loaded strongly onto their respective group factors, indicating that they are partially distinct from the general factor. In addition to openness, the content of decentering items reflect an ongoing awareness of thoughts and feelings and the ability to observe these experiences as separate from oneself. Likewise, whilst openness is embedded to a degree in committed action items, the content of these items also reflects flexible engagement in goal-directed behaviour.

Taken together, the general factor and decentering and committed action group factors observed here reflect components of the recently re-conceptualised three-part model of psychological flexibility: ‘open, aware, and engaged’ [11]. These newer summary terms have the advantage of being easier to use in clinical practice than the original six-part model [10]. Thus, the finding of a three-part structure in the present data is consistent with the evolving use of these terms as interpretive aids. Consistent with the wider, pragmatic philosophy behind Acceptance and Commitment Therapy, it is important to not be too rigid about these terms. As data accumulate, it is likely that the model and these terms will further evolve.

Zero-order correlation analyses indicated that the general factor was significantly associated with measures of social functioning, mental health, depression and pain intensity in the predicted directions. This pattern of results is consistent with a growing body of findings linking individual measures of psychological flexibility to positive pain-related outcomes in both cross-sectional and prospective studies [4, 5, 17]. Although significant, the correlation between the general factor and pain intensity was relatively weaker than the correlations between the general factor with social functioning, mental health and symptoms of depression. This pattern of findings is consistent with previous research and theory that suggest that pain intensity and facets of psychological flexibility may be only weakly related [8]. A non-significant relationship was observed between the general factor and physical functioning. The relationship between facets of psychological flexibility and indices of physical functioning has been somewhat inconsistent in the literature, with some studies reporting non-significant associations and others reporting weak associations [37, 38]. We presume that there are measurement challenges around the assessment of physical functioning that may hamper our ability to tap into either a general quality of daily functioning (or goal achievement) through asking about specifically ‘physical’ abilities. For example, a good quality of life is most likely achievable for many people without the ability to run, lift heavy objects, walk several flights of stairs or walk more than a mile without difficulty, to note sample items from the Short-Form Health Survey.

The present study draws on the study by Vowles and colleagues which used exploratory factor analyses to examine the structure of measures of psychological flexibility in chronic pain [22]. Both studies show that measures of individual psychological flexibility processes are highly related. The present study extends the work of Vowles and colleagues by using methods for robust hierarchical modelling. In particular, bifactor modelling has the advantage of allowing for simultaneous evaluation of the unidimensionality and multidimensionality of items, and explaining common variance across items rather than across latent variables, such as in higher-order factor modelling. Additionally, the present analyses were conducted on item responses versus questionnaire subtotal scores. The results from doing this indicate that a general factor reflecting openness underlies a number of current assessment measures, whilst measures of decentering and committed action are partially distinct from this general factor.

To date, a trend in research on facets of psychological flexibility has been to develop and validate self-report measures of individual component processes in isolation of the others. Whilst this is a logical approach, the present findings suggest that such a unidimensional focus may pose some limitations due to the presence of the general openness-related factor across a number of questionnaires. The observed saturation of items across the four questionnaires by this general factor indicates that the separate measures do not reliably measure the unique portions of the variance relating to theoretically distinct processes. Of course, the presence of an underlying factor which may obscure the measurement of purportedly distinct constructs is not an issue restricted to the psychological flexibility literature [39].

The current data reveal complexities in the measurement and conceptualisation of psychological flexibility. A single general factor that appears to reflect openness and distinct decentering and committed action group factors clearly emerge from the set of measures included here. However, the best and most efficient way to measure these components is not yet entirely clear and deserves further study. If there is practical or theoretical interest in the specific processes of psychological flexibility, as currently conceptualised, existing measures may need to be refined to include items that capture unique aspects of those processes that are distinct from openness. As data accumulate in the future, if findings consistently suggest that a single general factor underlies measures of purportedly distinct processes, the psychological flexibility model may need to be revised from its current multipart structure. It is important to consider that the current results are preliminary and, therefore, a decisive strategy for addressing these complexities is not possible at this time.

The results of this study should be considered in light of several limitations. One potential influence on the results obtained here arises from facets of psychological flexibility that were not well represented in the current data. Only a measure of committed action was used to assess the engaged process of psychological flexibility. Thus, the engaged component, which includes committed action and values-based action, is not fully represented in the larger item set here. Particularly, more development of measures of values-based action may be needed. A problem that is encountered in clinical practice is that uncovering a practical sense of peoples’ true values can require considerable training, shaping and the addressing of emotional and cognitive barriers. These kinds of challenges seem to limit current assessment methods for values [40].

Although we have previously shown that there is ‘self-as-context’ content in the decentering measure used here [21], these items are only minimally present. In their exploratory factor analyses, Vowles et al. reflected a process related to self with a measure of self-compassion [22]. This includes a conceptualisation of self that comes from a tradition that is different from behaviour analysis and functional contextualism, the roots of psychological flexibility. There appear to be no published measures that conceptualise the self in terms of psychological flexibility. Certainly, self-as-context requires more attention as a part of the wider model, as does present-focussed awareness.

Another limitation is the exclusive reliance on self-report measures. Shared method variance may have contributed to some degree to the magnitude of relations observed. Further development of measures that do not rely exclusively on self-report, such as implicit assessment procedures [41], may facilitate future investigation into psychological flexibility. It is important to clarify that the model of psychological flexibility examined here is made up of a mix of item content, with some items positively keyed and some negatively keyed and reversed prior to the analyses. With the exception of items from the Cognitive Fusion Questionnaire which are not typically reversed prior to analysis, each item set was scored in the direction that is consistent with the design of the measure from which it is obtained. Perhaps most importantly, this was a cross-sectional study and, therefore, inferences about the causal relationships that may lay behind the correlations observed between processes of psychological flexibility and patient-reported outcomes cannot be made. Analyses for the present study were conducted on pre-treatment data only. Therefore, future research should examine whether a similar factor structure emerges for assessment data collected on the measures reported here following treatment. Finally, the sample consisted of individuals with long-standing pain with significant distress and disability attending an intensive interdisciplinary treatment programme. Thus, future research is needed to test the generalisability of the findings to individuals with chronic pain with presentation features that differ from the current sample and to individuals with other health conditions.

Despite these limitations, this study is the first to investigate the structure of measures of psychological flexibility by evaluating lower-order, higher-order and bifactor models of measures of processes from within this model in a large sample of patients with chronic pain. Support was found for a general factor reflecting openness that underlies variability in items across measures of a number of processes of psychological flexibility. In addition to this general factor, distinct decentering and committed action group factors emerged in the data. Future research is needed to determine the most useful means by which the presence of the general factor can be reflected in the measurement and theory of psychological flexibility.
